# A Trp53^fl/fl^Pten^fl/fl^ mouse model of undifferentiated pleomorphic sarcoma mediated by adeno-Cre injection and *in vivo* bioluminescence imaging

**DOI:** 10.1371/journal.pone.0183469

**Published:** 2017-08-25

**Authors:** Marisa R. Buchakjian, Nicole M. Merritt, Devon L. Moose, Adam J. Dupuy, Munir R. Tanas, Michael D. Henry

**Affiliations:** 1 Department of Otolaryngology – Head and Neck Surgery, University of Iowa Hospitals & Clinics, Iowa City, Iowa, United States of America; 2 Department of Molecular Physiology and Biophysics, University of Iowa Carver College of Medicine, Iowa City, Iowa, United States of America; 3 Holden Comprehensive Cancer Center, University of Iowa Hospitals & Clinics, Iowa City, Iowa, United States of America; 4 Department of Pathology, University of Iowa Hospitals & Clinics, Iowa City, Iowa, United States of America; 5 Department of Anatomy and Cell Biology, University of Iowa Carver College of Medicine, Iowa City, Iowa, United States of America; Johns Hopkins University, UNITED STATES

## Abstract

Genetic mouse models of soft tissue sarcoma provide critical insights into disease pathophysiology, which are oftentimes unable to be extracted from human tumor samples or xenograft models. In this study we describe a mouse model of soft tissue sarcoma mediated by adenoviral-Cre recombinase injection into Trp53^fl/fl^/Pten^fl/fl^ lox-stop-lox luciferase mice. Injection of adenovirus expressing Cre recombinase, either subcutaneously or intramuscularly in two experimental groups, results in viral infection and gene recombination with 100% penetrance within the first 24 hours following injection. Luciferase expression measured by real-time bioluminescence imaging increases over time, with an initial robust increase following viral injection, followed by a steady rise over the next several weeks as primary tumors develop and grow. Intramuscular injections were more commonly associated with evidence of systemic viral distribution than subcutaneous injections. All mice developed soft tissue sarcomas at the primary injection site, with histological examination identifying 93% of tumors as invasive pleomorphic sarcomas based on microscopic morphology and immunohistochemical expression of sarcoma markers. A lymphocytic infiltrate was present in 64% of the sarcomas in this immunocompetent model and 71% of tumors expressed PD-L1. This is the first report of a viral-Cre mediated *Trp53/Pten* mouse model of undifferentiated pleomorphic sarcoma. The bioluminescence imaging feature, along with high penetrance of the model and its immunological characteristics, makes it suited for pre-clinical studies of soft tissue sarcoma.

## Introduction

Soft tissue sarcomas pose a significant clinical challenge, with approximately 12,000 new diagnoses and an estimated 4,700 sarcoma-related deaths per year [1]. Soft tissue sarcoma is a malignant mesenchymal neoplasm that is able to arise from many different tissue types and may occur in nearly any location in the body. Sarcoma may develop from muscle, fat, cartilage, connective tissue, nerves, blood vessels, and bone, and there is considerable heterogeneity between tumor types. Over 60 clinicopathologic subtypes of sarcoma have been described in the literature [2]. The most frequently diagnosed soft tissue sarcoma in adults is undifferentiated pleomorphic sarcoma (UPS), with the most common locations being the extremities and trunk. The tumor initiating cell of UPS is not currently known; it is considered by pathologists and clinicians to be a diagnosis of exclusion [3]. Histologically, UPS demonstrates spindle cell morphology, mitotic activity, nuclear pleomorphism, and areas of tumor necrosis; immunoreactivity for myogenic markers such as smooth muscle actin (SMA), and desmin may be present in UPS, but they do not fully exhibit skeletal muscle or smooth muscle differentiation [4].

Soft tissue sarcomas are broadly classified as either tumors with simple karyotypes, which are defined by characteristic chromosomal translocations or gene amplifications and few other cytogenetic alterations, or those with complex karyotypes containing multiple cytogenetic alterations [5]. Soft tissue sarcomas harboring complex karyotypes comprise approximately two-thirds of all sarcomas, including UPS [6]. One of the most common genetic alterations in sarcomas with complex karyotypes is tumor protein 53 (*TP53*) inactivation, either via somatic mutation, deletion, or alterations in *TP53* pathway genes such as *CDKN2A* and *MDM2* [5,7]. Loss of p53 is known to cause global genetic instability and promote additional pro-tumorigenic mutations due to its critical role in regulating DNA repair, cell cycle, programmed cell death, and cellular senescence [8]. *TP53* appears to play a particularly important role in sarcomagenesis; 25–30% of tumors that develop in patients with germline *TP53* mutations (patients with Li-Fraumeni syndrome) are sarcomas, specifically soft tissue sarcomas and osteosarcomas [9].

Phosphatase and tensin homolog (PTEN) is a well-characterized tumor suppressor protein involved in regulating the phosphoinositide 3-kinase (PI3K) pro-growth pathway. PTEN lies upstream of the phosphoinositide 3-kinase (PI3K)-AKT-mammalian target of rapamycin (mTOR) axis, which has broad roles in directing cellular growth, metabolism, division, senescence, and migration [10]. Loss of PTEN expression has been described in approximately 29% of soft tissue sarcomas [11]. Notably, the PI3K pathway is thought to be activated in greater than 80% of rhabdomyosarcomas, and both TP53 and PTEN function are lost in approximately 67% of leiomyosarcomas [12,13].

Mouse models of cancer provide critical insights into disease mechanisms, and there is a need for translational sarcoma models that recapitulate human disease and provide realistic opportunities for tumor characterization and pre-clinical testing. In this study, we sought to characterize the role of concurrent *Trp53* and *Pten* deletion in a mouse model of sarcoma. This model is unique in promoting the combined homozygous loss of the *Trp53* and *Pten* alleles with the advantage of temporal and spatial control over recombination. We describe a viral-mediated inducible mouse model of soft tissue sarcoma based on somatic induction and tissue-specific homozygous loss of *Trp53* and *Pten*. Importantly, this model is coupled with Cre-dependent luciferase expression to facilitate *in vivo* longitudinal, noninvasive bioluminescence imaging (BLI) of gene recombination and tumor formation [14]. This model closely recapitulates early, stochastic inactivation of *TP53* and *PTEN* in human sarcomas, and allows for the neoplastic steps of driver mutation accumulation, tumor initiation, growth, and proliferation to fully transpire.

Development of this model supplies a new tool for following sarcomagenesis driven by inactivation of *Trp53* and *Pten*, providing opportunities to better understand tumor initiation and progression. The majority of mouse models of soft tissue sarcoma described in the literature focus on xenograft or orthotopic models, which bypass the critical steps of tumor formation and initial growth and do not allow for the study of sarcomagenesis with an intact immune response. Researchers have also developed genetic models of sarcoma focusing on either global or tissue-specific gene loss to promote tumor growth [15]. *Trp53* germline global knockout models of sarcoma have been described, with tumors including undifferentiated sarcomas, hemangiosarcomas, and osteosarcomas, in addition to other primary tumors, however these models are limited by longevity of the animals and substantial tumor burden [16]. A recent study of conditional heterozygous loss of *Trp53* and *Pten* in smooth muscle via the transgelin promoter demonstrated growth of high-grade UPS, leiomyosarcomas, and carcinosarcomas via inappropriate activation of the NOTCH differentiation pathway [13]. Homozygous loss of *Trp53* and *Pten* in mouse adipose tissue has also been demonstrated to result in multiple subtypes of liposarcoma, including well-differentiated, de-differentiated, myxoid/round cell, and pleomorphic liposarcoma [17].

Viral-mediated models are a more recent advancement in transgenic mouse model technology. Advantages to this approach include the ability to more readily control temporal and spatial induction of allelic recombination. A model of high-grade sarcoma with myofibroblastic differentiation based on intramuscular adenoviral-Cre injection into *Trp53/Kras* conditional mutant transgenic mice was described by Kirsch et al. [18]. In this study, adenovirus (Ad) containing a Cre recombinase expression vector was injected into the extremity muscles of transgenic mice, resulting in knock-out of *Trp53* and activation of oncogenic mutant *Kras* (LSL-KRas^G12D^ Trp53^flox/flox^). Sarcomas developed in 90% of the extremities injected, and microscopic lung metastases were detected in 20% of mice with large primary tumors (>2cm) [18]. This model demonstrated the ability of Ad-Cre to promote intramuscular allelic recombination and soft tissue sarcoma formation in an oncogenic *Trp53/Kras* genetic background, and highlighted the utility and convenience of viral-mediated mouse models. Mito et al. went on to further characterize the LSL-KRas^G12D^ Trp53^flox/flox^ extremity tumors, confirming via cross species genome analysis that mouse tumors shared statistically-significant gene enrichment as well as histology with human UPS [19]. This model also has a 40% metastatic rate to the lung after primary tumor resection to allow for additional time for metastases to develop [18–20]. The metastatic potential of UPS to the lung in the LSL-KRas^G12D^ Trp53^flox/flox^ model appears to be at least in part driven by mir-182 expression [20]. In a similar background model (LSL-KRas^G12D^ Trp53^flox/flox^), DuPage et al. induced sarcomagenesis via intramuscular lentiviral injection, with viral expression vectors containing Cre recombinase alone or with T-cell antigens fused to the carboxy terminus of firefly luciferase in a study of cancer immunoediting [21].

The aim of the current study was to develop a viral-mediated mouse model of soft tissue sarcoma that also includes *in vivo* BLI capabilities. BLI allows for real-time, longitudinal imaging of allelic recombination and transgene expression, and when tracked over time serves as a marker for tumor formation and growth. This provides the opportunity for quantitative surrogate measurements of tumor growth without requiring interval sacrifice of valuable animals from the study cohort. Our mouse model harbored Trp53^fl/fl^ and Pten^fl/fl^ alleles, as well as a lox-stop-lox ROSA26 luciferase allele. Expression of Cre recombinase resulted in loss of expression of *Trp53* and *Pten* while promoting luciferase expression. Ad-Cre recombinase was injected either subcutaneously or intramuscularly to compare results between the two groups. The mice were then followed longitudinally with *in vivo* BLI as primary tumors developed and grew. This model has an advantage over non-BLI viral transgenic models in that viral infection and gene recombination are readily identified and evaluated over time, allowing for more accurate and less invasive assessment of the neoplastic process.

## Materials and methods

### Mouse strains and genotyping

All animal procedures were performed with approval from the University of Iowa Institutional Animal Care and Use Committee, protocol number 1302027. We crossed homozygous C57BL/6J-Tyr^c-2J^/J ROSA26 LSL-Luc Pten^fl/fl^ mice with heterozygous B6.129P2-Trp53^tm1Brn^/J (Jackson Laboratory) [22]. The C57BL/6J-Tyr^c-2J^/J ROSA26 LSL-Luc Pten^fl/fl^ are on an albino C57BL/6J background. The B6.129P2-Trp53^tm1Brn^/J mice are on a mixed C57BL6/129 background. The resultant intercross was extensively backcrossed (n = 6) onto C57BL/6J-Tyr^c-2J^. Both the *Pten* and *Trp53* floxed alleles and Tyr^c-2J^ (for albino coat color to facilitate BLI) were bred to homozygosity. Offspring were genotyped by PCR for the presence of the ROSA26 LSL-Luc, Trp53^fl/fl^, and Pten^fl/fl^ alleles using gene-specific primers [22]. Two to three-month old mice were used for the study, with 38% female and 62% male mice. Three control mice and ten experimental mice were included in each injection group for a total of 26 mice examined.

### Adenovirus injection

C57BL6 ROSA26 LSL-Luc Trp53^fl/fl^ Pten^fl/fl^ mice were first anesthetized in a chamber using 3% isoflurane in order to facilitate accurate injections. Ad5 CMV-eGFP (control animals) or Ad5 CMV-Cre recombinase eGFP (experimental animals) obtained from The University of Iowa Viral Vector Core was injected either subcutaneously overlying the right posterior flank or intramuscularly into the posterior right quadriceps. Injections were performed using 1x10^8^ pfu in a total of 20μL injection volume (viral stocks were diluted to final concentration using 1x phosphate-buffered saline). Successful injections were confirmed either by observing a wheal in the subcutaneous group or by manual stabilization of the quadriceps muscle and palpation of the femur with the needle tip prior to injection in the intramuscular group.

### Bioluminescence imaging

All BLI was performed using an Ami X imager from Spectral Instruments Imaging (Tucson, AZ). For *in vivo* mouse imaging, the animals were first anesthetized in a chamber with 3% isoflurane. They were then injected intraperitoneally with 150mg/kg D-luciferin (VivoGlo^™^, Promega, Madison, WI) and placed on the imaging platform while maintaining inhalational anesthetic. Mice were imaged 5 minutes after D-luciferin injection for a total of 5 minutes exposure time, first prone and then repositioned to left lateral decubitus, with a field of view of 25cm and an object height of 1.5cm. AMIView Imaging Software was used to define the region of interest on the image in order to measure BLI values. For whole body BLI measurements a region of interest was drawn to encompass the entire mouse. For leg-only BLI values a region of interest was centered on the right posterior leg and flank, and for abdomen-only values a region of interest was centered between the upper and lower extremities. Comparative BLI figures are presented on a log scale with a threshold of 2.0x10^5^ and maximum value of 1.1x10^7^. *Ex vivo* BLI was performed by imaging the mice and dissected tissues for an additional 5 minutes immediately post-necropsy. For *ex vivo* images, euthanasia was performed and the primary tumor was quickly removed to allow for imaging of the remainder of the animal and to evaluate for lesions with low BLI signal. BLI values as measured by photon flux (photons/sec/cm^2^/sr) were obtained using AMIView Imaging Software, version 1.5.0.

### Mouse examinations

All mice were examined and weighed weekly. Examination included palpation of the leg/flank and abdomen and caliper measurements of primary tumors. *In vivo* BLI was performed on the complete cohort biweekly and on individual animals as needed for survival surgeries and necropsies. Survival surgeries were performed by anesthetizing the mice with 3% isoflurane throughout the duration of the procedure. Microsurgical scissors and forceps were used to dissect the tumor from the surrounding tissue, and pressure was applied for hemostasis. Surgical clips were used to reapproximate the skin and were removed one week postoperatively. Mice were monitored daily for one week postoperatively. Euthanasia, *ex vivo* imaging, and dissections were performed when mice reached pre-determined end points, including 20% loss of starting body weight, primary tumor size >2cm, decreased mobility, lethargy/lack of grooming, or other gross morbidity. Control mice were also euthanized at the completion of the study. Primary tumors and other appropriate tissues were collected and incubated in 10% neutral buffered formalin (Research Products International Corp., Mount Prospect, IL) at 4°C for 24–48 hours, transferred to 70% ethanol, and embedded in paraffin.

### Histopathology

The following antibodies were utilized for immunohistochemistry on formalin fixed paraffin embedded tissue. Anti-pancytokeratin (mouse monoclonal AE1/AE3; catalog # NB600-1322) was obtained from Novus Biologicals (Littleton, CO, USA). Anti-S100 protein (rabbit polyclonal; catalog # Z0311) was obtained from Dako (Carpinteria, CA, USA). Anti-SMA (mouse monoclonal 1A4; catalog # A2547) was obtained from Sigma-Aldrich (St. Louis, MO, USA). Anti-desmin (mouse monoclonal D33; catalog # M0760) was obtained from Dako. Anti-myogenin (mouse monoclonal F5D; catalog# M3559) was obtained from Dako. Anti phospho-AKT Ser473 (rabbit monoclonal D9E; Cell Signaling Technology catalog #4060 (Danvers, MA, USA). Anti-PTEN (rabbit monoclonal D4.3 XP; catalog# 9188) was obtained from Cell Signaling Technology. Anti-CD45 (rabbit polyclonal; catalog# 10558) was obtained from Abcam (Cambridge, MA, USA). Anti-PD-L1 (rabbit polyclonal; catalog# 17952-1-AP) was obtained from Proteintech (Rosemont, IL, USA). Dilutions utilized for immunohistochemistry are as follows pan-cytokeratin (1:1600), S100 protein (1:4000), SMA (1:2000), desmin (1:50), myogenin (1:50), phospho-AKT (1:50), PTEN (1:50), CD45 (1:100), and PD-L1 (1:50). Horseradish peroxidase-conjugated secondary antibodies were obtained from Dako.

### PCR analysis

Genomic DNA was isolated from formalin-fixed paraffin embedded tumors using the QIAamp^®^ DNA FFPE Tissue Kit (Qiagen, Germantown, MD) or tail snips using the DNeasy^®^ Blood & Tissue Kit (Qiagen, Germantown, MD). The samples were lysed overnight at 56°C and subsequent steps were performed according to the manufacturer’s instructions. To assay for recombination of the floxed *Pten* allele, DNA isolated from the tumor was amplified using primers described previously [23]. *Trp53* recombination was evaluated using primers flanking the 2^nd^ and 11^th^ exon, as previously reported [24].

### Statistical analysis

All statistical analysis was performed with GraphPad Prism software 7.0. For BLI analyses, data were transformed using the natural log and then a two-way ANOVA was performed with multiple comparisons and a Bonferroni correction. Data for tumor latency was analyzed using a non-paired Welch’s t-test, and the Spearman test was used to correlate BLI with tumor mass and volume. Clinical and genomic data for all TCGA soft tissue sarcomas were obtained from the cBioPortal (http://www.cbioportal.org). Only samples with clinical as well as summarized copy number and somatic mutation data were analyzed. Four tumor classes were considered for each type of tumor: *TP53* mutant only, *PTEN* mutant only, *TP53* and *PTEN* mutated, or *TP53* and *PTEN* wild type. The frequency for each class was compared for UPS versus all other forms of soft tissue sarcoma using a Chi-squared statistic (degrees of freedom = 3).

## Results

### *Trp53/Pten*-floxed mice injected with Ad CMV-Cre demonstrate a longitudinal increase in BLI signal

**Fig 1:**
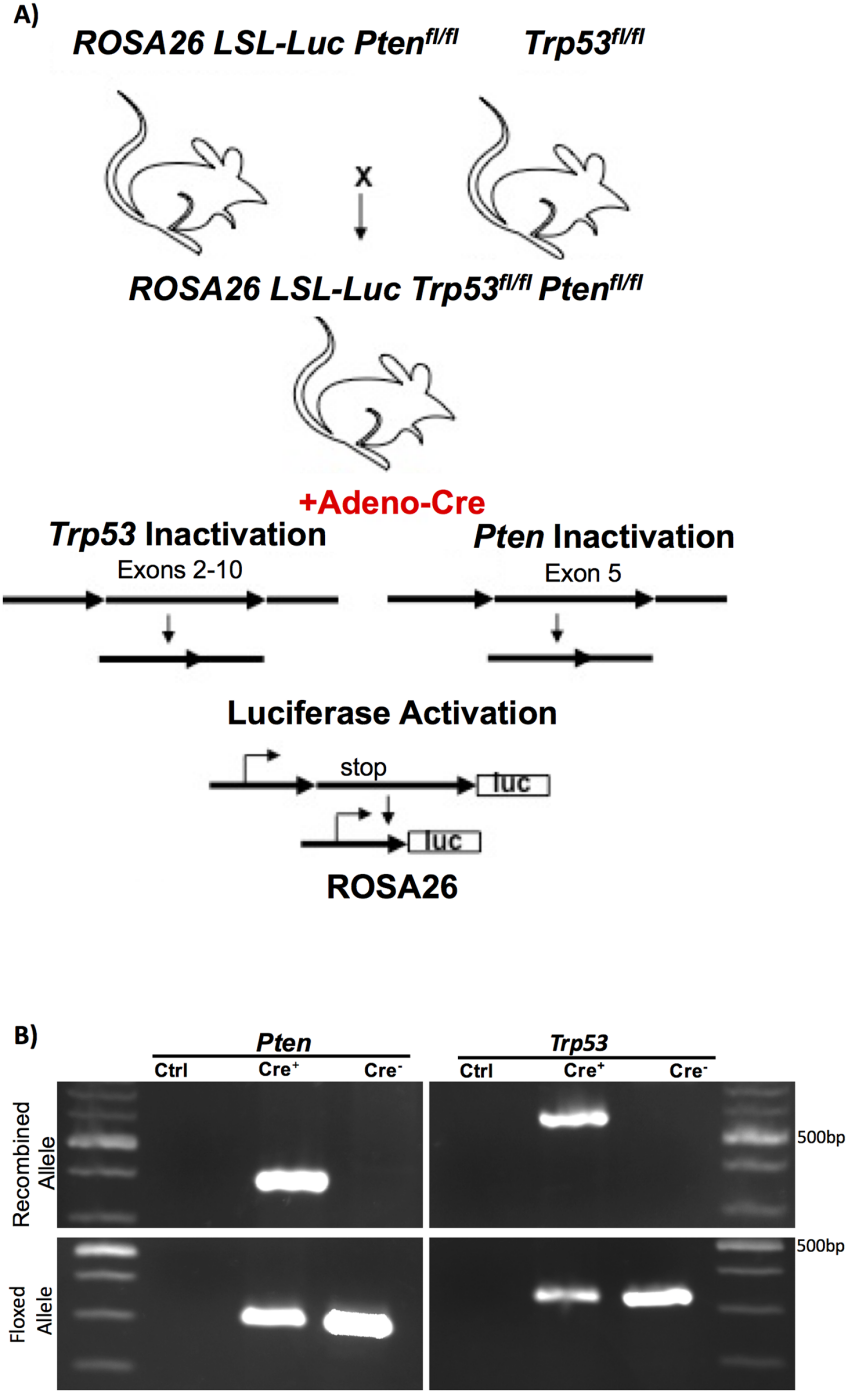
Generation of *Trp53/Pten* mice associated with luciferase expression. A) ROSA26 Lox-stop-Lox Luciferase, Pten^fl/fl^ male mice were crossed with Trp53^fl/fl^ female mice to generate offspring with ROSA26 LSL-Luc, Pten^fl/fl^, and Trp53^fl/fl^. Ad with a Cre recombinase expression vector was injected either subcutaneously over the right leg/flank or intramuscularly into the right quadriceps to promote somatic recombination. Somatic expression of Cre recombinase promoted expression of luciferase and homozygous knockout of *Pten* and *Trp53*. B) PCR from DNA isolated from either a tumor of Cre injected mice (Cre+) or from the tail of a non-injected mouse (Cre-) (Ctrl is a primer only control) to assay for the presence of recombination and floxed *Pten* or *Trp53*.

In this study we examined the consequence of temporally and spatially controlled *Trp53* and *Pten* deletion by somatic Cre recombinase expression on sarcoma formation. Review of the TCGA database indicates that co-mutation of *TP53* and *PTEN* occurs more frequently (66.7%; n = 21) in UPS as compared to all other soft tissue sarcomas (43.4%; n = 221) (Chi-squared = 28.108, df = 3, p<0.0001), indicating that this combination of mutations is frequently observed in human UPS. The experimental mice were created by crossing ROSA26 lox-stop-lox luciferase, Pten^fl/fl^ and Trp53^fl/fl^ mice together, to create offspring with three homozygous floxed alleles (Fig 1A). Ad CMV-Cre recombinase or Ad CMV-eGFP were injected either subcutaneously (SQ) over the right flank or intramuscularly (IM) into the right quadriceps. The SQ compartment is composed of adipocytes, fibroblasts, and their precursors. The IM compartment is composed of skeletal muscle cells and their precursors (e.g. satellite cells). Since it is unclear what the tumor initiating cell is for many sarcomas, we injected both the SQ and IM compartments. Adenoviral infection and Cre recombinase expression promoted allelic recombination, including expression of luciferase to accommodate longitudinal BLI as well as homozygous loss of *Trp53* and *Pten* tumor suppressors (Fig 1B). We noted that the floxed alleles for both *Pten* and *Trp53* are still detectable by PCR in the tumor tissue. This could reflect that both alleles are not deleted in all of the cancer cells and/or that there is stromal and immunologic contamination in the tumor contributing to this signal. To partially address this, we performed immunohistochemistry for PTEN which revealed loss of expression in all of the sarcomas evaluated (Table 1 and S1 Fig), thus, at least PTEN appears to be deleted in all of the tumor cells.

**Table 1:** Pathological features of *Trp53/Pten* sarcomas.

Tum	Ker	S100	SMA	Des	Myo	PTEN	PDL1	Lymph	Type
1	-	-	-	-	-	-	-	+	UPS
2	-	-	V+	-	-	-	D+	+	UPS
3	-	-	V+	-	-	-	F+	-	UPS
4	-	-	V+	R+	-	-	V+	+	UPS
5	-	-	V+	R+	V+	-	F+	-	PRMS
6	-	-	V+	V+	-	-	F+	+	UPS
7	-	-	V+	V+	-	-	D+	+	UPS
8	-	-	D+	-	-	-	V+	-	UPS
9	-	-	-	-	-	-	-	+	UPS
10	-	-	-	-	-	-	-	-	UPS
11	-	-	-	-	-	-	F+	-	UPS
12	-	-	-	-	-	-	V+	+	UPS
13	-	-	-	-	-	-	F+	+	UPS
14	-	-	-	-	-	-	-	+	UPS
Total	0% (0/14)	0% (0/14)	50% (7/14)	29% (4/14)	7% (1/14)	0% (0/14)	71% (10/14)	64% (9/14)	UPS (93%) PRMS (7%)

**Fig 2:**
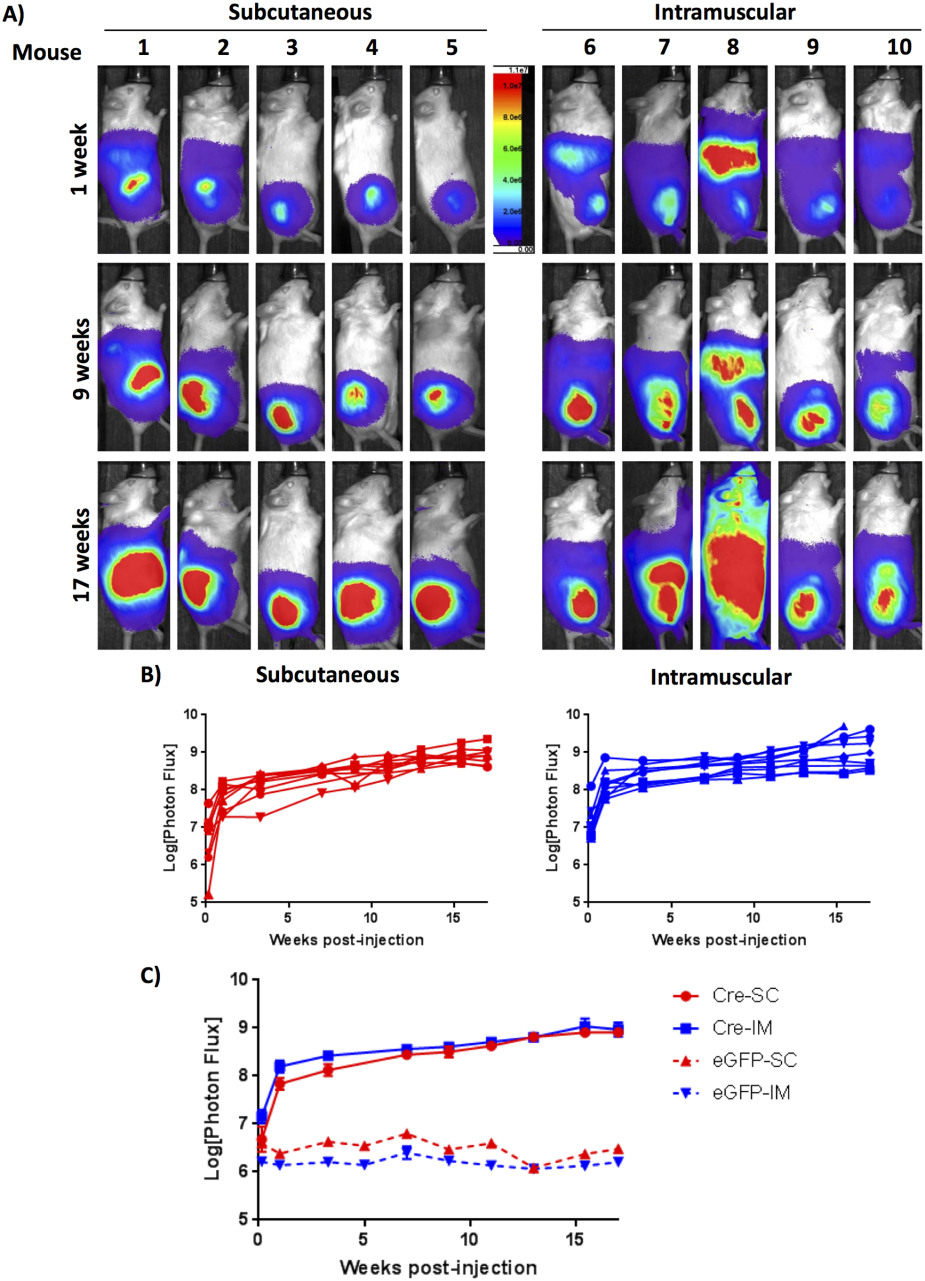
*Trp53/Pten* mice injected with Ad CMV-Cre demonstrate a longitudinal increase in BLI signal. A) BLI of *Trp53/Pten* mice injected either SQ or IM with Ad CMV-Cre. Mice were injected with intraperitoneal D-luciferin and imaged using an Ami X imager. Shown are representative images of five mice from each cohort at 1 week, 9 weeks, and 17 weeks post-viral injection, demonstrating a steady increase in luciferase signal over time. B) Quantified whole-body *in vivo* BLI values as measured in photons/sec/cm^2^/sr are shown for individual mice for each injection group (n = 9), and compared between the two injection groups C), highlighting similar luciferase expression kinetics and values between SQ- and IM-injected animals. The difference between SQ and IM injection was statistically significant for day 1 post-injection (p = 0.045), but not thereafter. From the 2way ANOVA, overall variance due to injection method was statistically significant (p = 0.0019). In comparison to the first day post-injection, all values for both injection methods were significant (p<0.0001).

To evaluate for luciferase expression as a marker of recombination, mice were injected with D-luciferin and *in vivo* BLI was performed one day post-viral exposure, 1 week post-exposure, and biweekly thereafter. Ad CMV-eGFP control mice, as expected, did not demonstrate BLI signal (Panels A and B in S2 Fig). Mice injected with Ad CMV-Cre via SQ or IM administration showed a robust and reproducible increase in whole-body BLI signal, with a substantial increase in BLI signal within the first week, likely representing viral infection and initial recombination events; this was followed by a steady increase in luciferase expression at the site of injection over subsequent weeks as depicted by BLI signal at weeks 1, 9, and 17 across five representative mice from each cohort (Fig 2A). Whole-body BLI is also demonstrated quantitatively for individual mice in each injection group in Fig 2B, with comparable expression levels and growth kinetics across animals (n = 9 for each group). BLI signal is compared between the two injection groups in Fig 2C, highlighting similar luciferase expression at the primary injection site for the SQ and IM injected cohorts. Importantly, 100% of *Trp53/Pten* mice injected with Ad CMV-Cre demonstrated luciferase expression by BLI at the site of injection, whereas all mice injected with the control virus Ad CMV-eGFP exhibited only background levels of bioluminescence signal for the duration of the study (Fig 2C). Thus, injection of Ad CMV-Cre either subcutaneously or intramuscularly is able to promote reliable viral infection and allele recombination.

### IM and SQ injection of Ad CMV-Cre results in abdominal BLI signal

**Fig 3:**
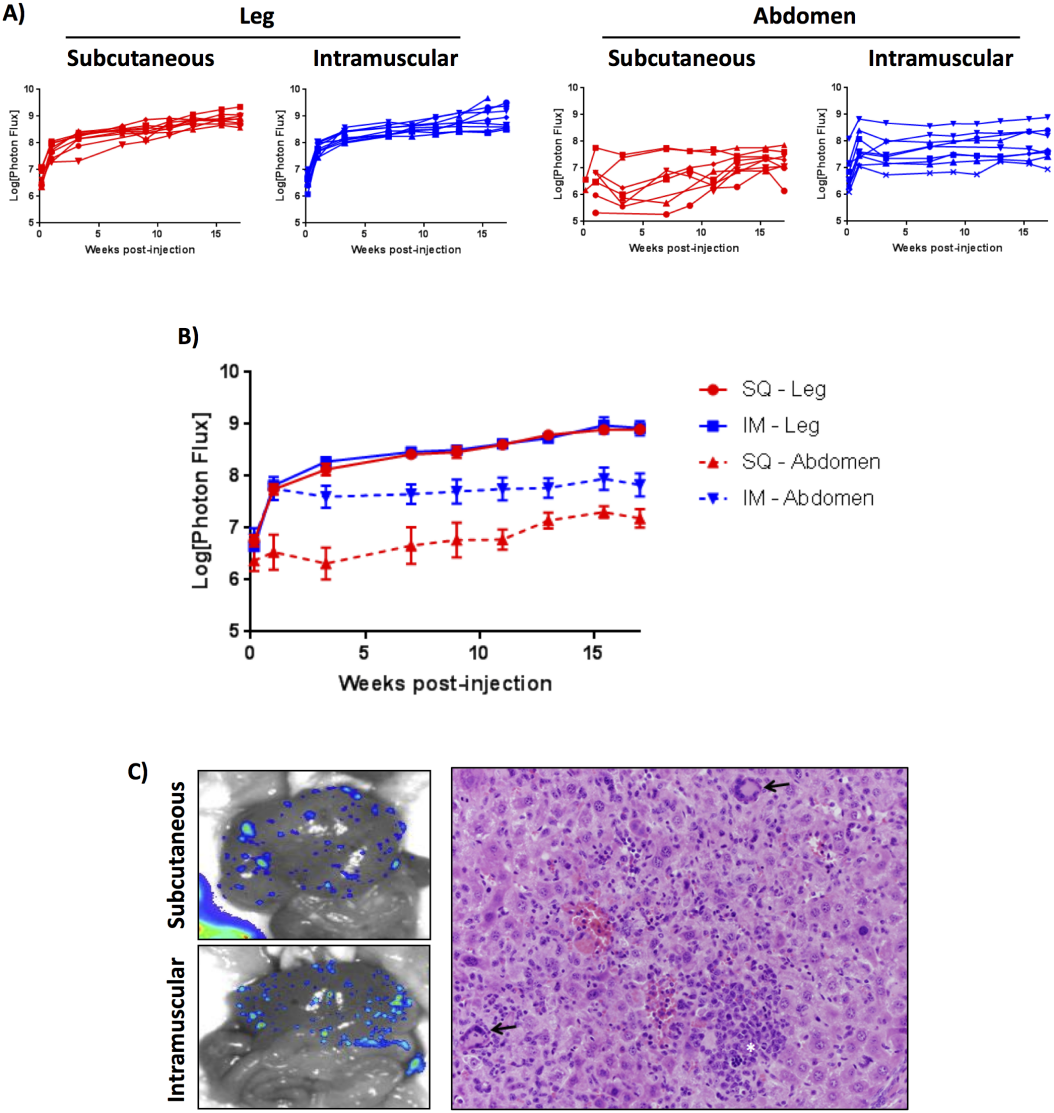
IM-injected Ad CMV-Cre results in increased systemic viral spread. A) Quantified leg-only or abdomen-only BLI values are shown for individual Trp53^fl/fl^Pten^fl/fl^ mice injected either SQ or IM with Ad CMV-Cre. B) Comparisons between injection cohorts demonstrate lower non-specific signal in the abdomen with SQ injection. There was no significant difference in luciferase signal between the SQ- or IM-injected legs; however, there is a significant difference between SQ and IM abdomen injections for week 1 and beyond (p<0.05). The difference in comparison to the first day post-injection was statistically significant for both SQ (p<0.002) and IM (p<0.0001) leg injections as well as IM abdominal BLI (p<0.02). No significant difference was found for SQ abdominal BLI in comparison to day 1. C) *Ex vivo* BLI images of livers from one SQ- and one IM-injected mouse, depicting multifocal low-level recombination and luciferase expression in the livers of mice with abdominal BLI signal, suggesting systemic viral spread and infection (left panels). Histological sections of liver demonstrated extramedullary hematopoiesis, noted with arrows, but no neoplasm was identified (right panel).

Although whole-body BLI did not significantly differ between the SQ- and IM-injected *Trp53/Pten* cohorts, we noticed obvious differences in the BLI signal in the region of the abdomen when the two injection methods were compared. Rather than measuring whole-body BLI, a region of interest for luciferase signal was drawn either over the right leg and flank or isolated over the abdomen. Results are shown in Fig 3A for individual mice from each group and in Fig 3B between SQ and IM groups. These data demonstrate a near identical BLI signal at the primary viral injection site when measuring the leg in isolation. However, when examining BLI signal in the abdomen, the IM-injected mice had significantly greater abdominal luciferase expression compared to the SQ-injected mice (Fig 3A and 3B). The difference in abdominal BLI signal between SQ and IM animals was evident as early as 24 hours post-injection, increased by the first week, and then remained fairly stable over time. Abdominal signal was seen in 100% of IM-injected mice, and appreciable abdominal signal was seen in only 20% of SQ-injected mice (Fig 3A and further shown in Fig 2A, mice 6–10 compared to mice 1–5, including the two SQ injected mice with abdominal signal). These data suggest that IM injection of Ad likely results in increased systemic absorption and viral infection in regions of the body distinct from the initial injection site. *Ex vivo* BLI of livers during necropsy revealed that the source of BLI signal in the abdomen is likely secondary to multiple areas of low-level recombination within the liver (Fig 3C, left panels). This result was independently confirmed with another set of *Trp53/Pten* mice IM-injected with both Ad CMV-Cre and control Ad CMV-eGFP virus, demonstrating both BLI *in vivo* as well as *ex vivo* BLI in the liver in IM Cre-injected mice and no appreciable luciferase signal in IM eGFP-injected mice (S2 Fig). Histologic analysis of livers with BLI signal demonstrated extramedullary hematopoiesis but no neoplasm (Fig 3C, right panel). These results are significant because increased systemic recombination and unanticipated BLI signal from the primary viral injection site results in an increased overall luciferase signal and background in the IM model, suggesting that the SQ *Trp53/Pten* model might provide improved localization of somatic recombination and decreased systemic BLI background.

### Trp53^fl/fl^Pten^fl/fl^ mice injected with Ad CMV-Cre form soft tissue tumors

**Fig 4:**
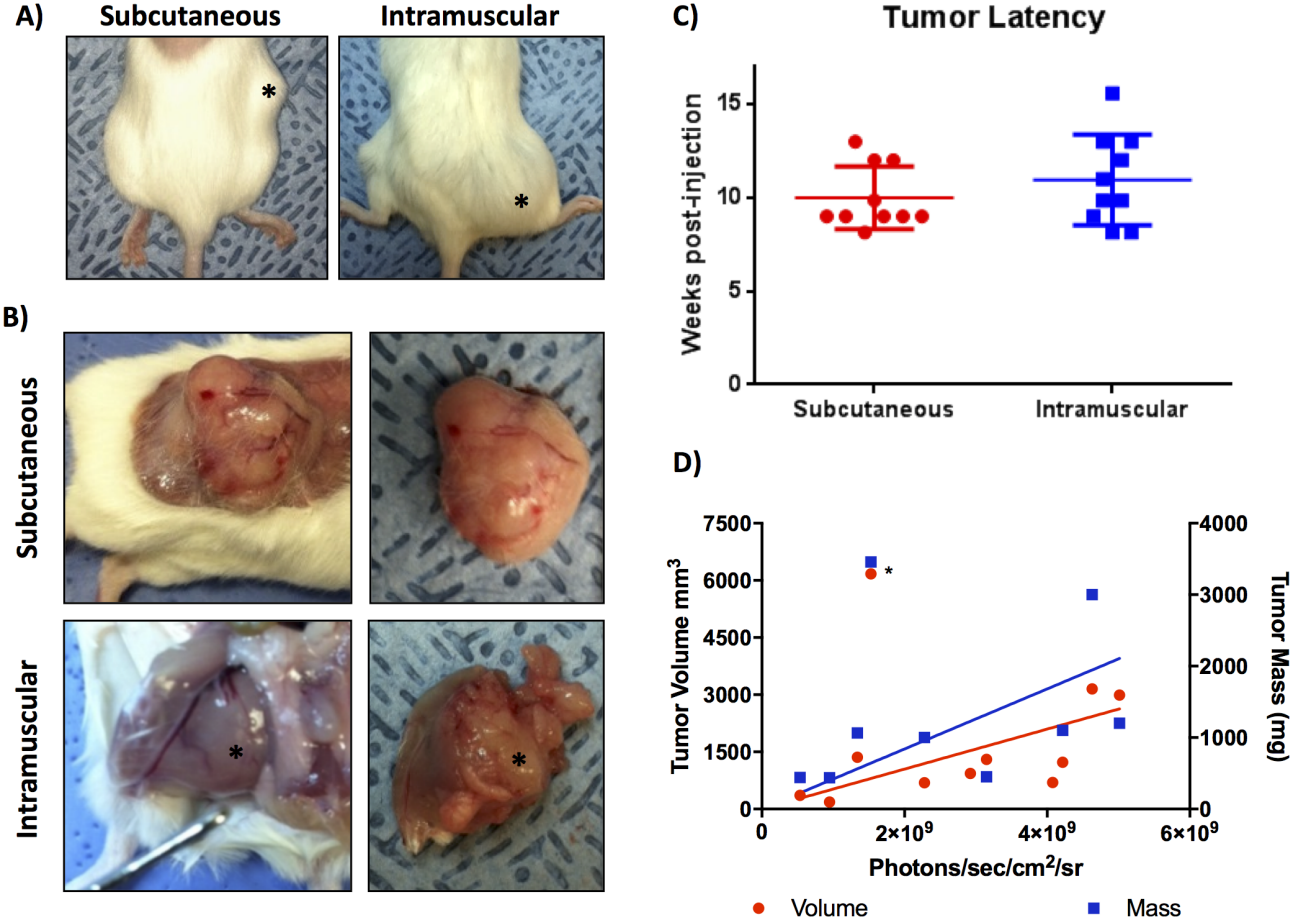
Trp53^fl/fl^Pten^fl/fl^ mice injected with Ad CMV-Cre form soft tissue tumors with 100% penetrance. A) Photographs of representative intact SQ and IM soft tissue tumors (*). B) Primary tumor dissections highlighting the superficial nature of tumors in the SQ-injected group compared to the deep, invasive tumors in the IM group, which develop within the native quadriceps muscle and adhere firmly to the surrounding femur and musculature. C) Palpable tumor latency as defined by time to earliest manual palpation of soft tissue tumor (p = 0.3201) D) Primary tumor mass and tumor volume with corresponding BLI measurements. Tumor mass (blue squares, right y-axis) measured after surgical excision and volume (red circles, left y-axis) measured by calipers are correlated with photon flux (X-axis); Spearman *r* = 0.857 and r = 0.733, respectively. Asterisk indicates one outlying tumor that was removed from the analysis.

Both SQ and IM injection of Ad CMV-Cre virus into Trp53^fl/fl^Pten^fl/fl^ mice resulted in primary soft tissue sarcomas arising at the site of injection with 100% penetrance. No tumors developed in control Ad CMV-eGFP mice. SQ viral injection over the right leg/flank resulted in development of relatively superficial, mobile, rubbery tumors, whereas IM viral injection resulted in deep, firm tumors within the muscle with apparent enlargement of the quadriceps muscle on physical exam (Fig 4A and 4B). The average tumor latency, as defined by the time of earliest manual palpation of tumor, was approximately 10 weeks for the SQ mice and 11 weeks for the IM mice, despite near-identical leg BLI measurements (Fig 4C). This suggests a minor discrepancy between the ability to manually detect the superficial subcutaneous tumors versus the more deeply-seated intramuscular tumors, however, differences in the kinetics of tumor development and growth cannot be excluded. Notably, tumor growth is detectable by BLI well in advance of detection by palpation (compare Figs 3A and 4C). Mice were euthanized when clinical end points were reached as described in the Materials and Methods section. Representative primary tumor dissections at the time of necropsy are shown in Fig 4B, highlighting the superficial, noninvasive nature of the SQ tumors compared to the deeply invasive IM tumors, which were embedded within native muscle and required aggressive dissection from the underlying skeleton and normal tissue. Fig 4D shows both primary tumor mass and volume as a function of BLI indicating that BLI signal is correlated with tumor size, although one very large tumor was an outlier in this analysis. The reason for this outlier is unclear, but limitations on BLI analysis are discussed below. Survival surgeries were attempted for three mice from the SQ group and two mice from the IM group to determine feasibility of primary tumor removal in an effort to allow for increased time for metastatic growth, however, not surprisingly the IM tumors were challenging to resect without significant morbidity to the animal. Interestingly, all mice that underwent primary resection did experience a recurrence at the primary tumor site, with an average recurrence time of 12.5 weeks for the SQ mice and only 3.9 weeks for the IM mice. Despite attempts at survival surgeries (n = 5) to extend observation time, no obvious metastases were detected by BLI in any of the experimental animals. These data highlight some major clinical differences between primary tumors in the SQ- and IM-injected Ad CMV-Cre *Trp53/Pten* mice, including more fixed and invasive tumors, longer latency to a palpable tumor, and greater morbidity in the IM group compared to SQ.

### Characterization of soft tissue tumors reveals they are predominantly undifferentiated pleomorphic sarcomas

**Fig 5:**
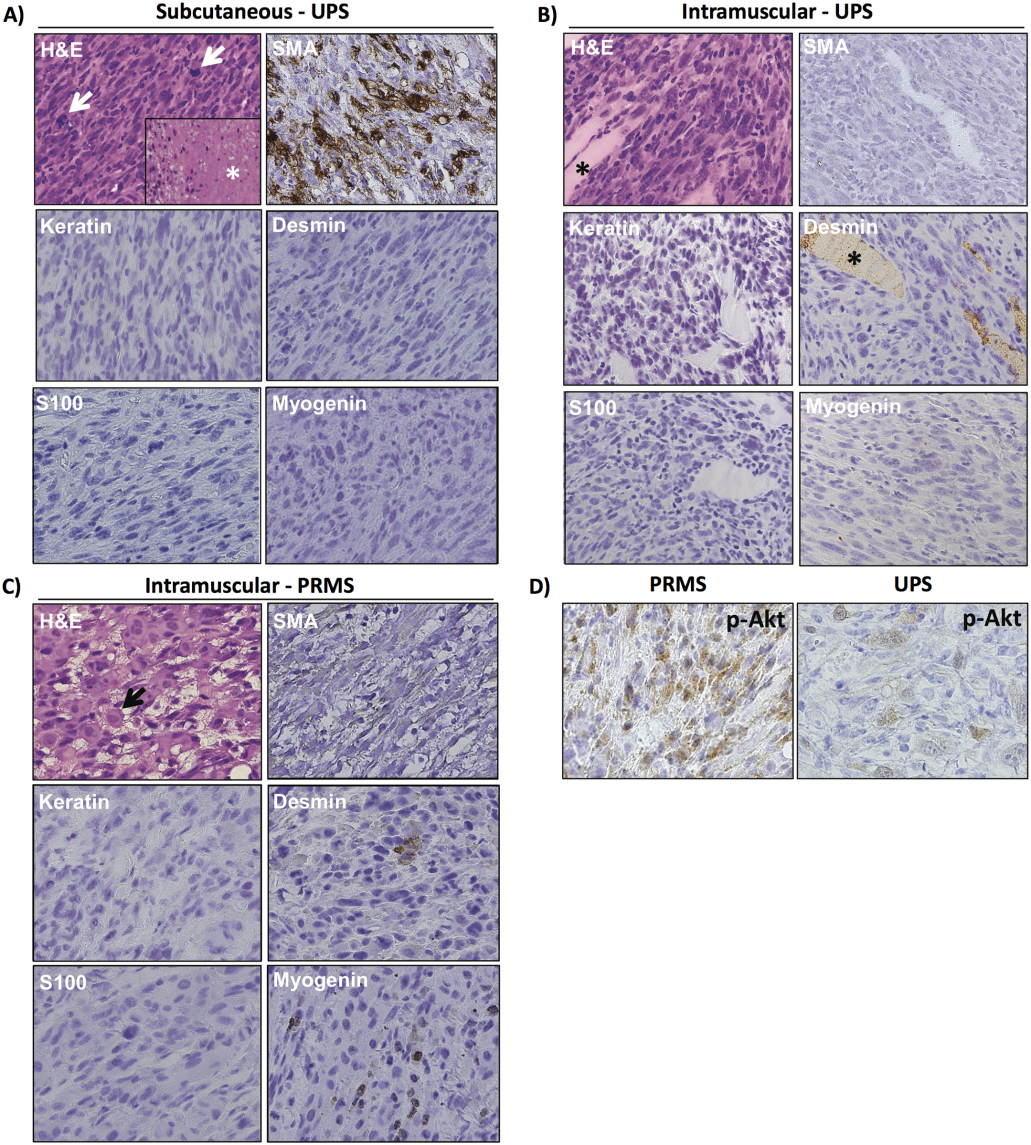
Trp53^fl/fl^Pten^fl/fl^ mice injected with Ad CMV-Cre form undifferentiated pleomorphic sarcoma. A) Histology and immunophenotype of UPS arising after SQ injection of Ad CMV-Cre. H&E section reveals a proliferation of markedly atypical spindle cells with numerous mitotic figures (arrows) as well as tumor necrosis (*). No line of differentiation was identified by the neoplasm’s light microscopic appearance or immunohistochemical profile, supporting classification as UPS. B) Histology and immunophenotype of typical UPS arising in skeletal muscle. The histological appearance and immunophenotype is similar to the subcutaneous UPS present in (A). SMA is variably positive in different examples, similar to human UPS. The asterisk highlights skeletal muscle fibers in the H&E section as well as the desmin stain, where they serve as an internal control. C) Histology and immunophenotype of PRMS. H&E section reveals a proliferation of markedly atypical spindle cells admixed with rounded rhabdomyoblasts (arrow). Skeletal muscle differentiation is confirmed by the identification of various foci expressing myogenin and desmin. D) Phospho-AKT was variably present. In some sarcomas, such as the PRMS tumor and some of the UPS tumors, phospho-AKT could be identified more diffusely (left panel). In other UPS tumors, it was more focal (right panel).

Primary tumors were collected from the SQ- and IM-injected Trp53^fl/fl^Pten^fl/fl^ mice at the time of necropsy, and tissue sections were analyzed for histology as well as by immunohistochemistry. Examination of histology from both SQ- as well as IM-arising tumors revealed findings consistent with UPS, demonstrating atypical spindle cells with no identifiable line of differentiation (Fig 5A and 5B). The tumors were described as having numerous mitotic figures (arrows), as well as areas of tumor necrosis (*). Tumors were further characterized by variable staining for smooth muscle actin (SMA), absent staining for keratin, S100, and myogenin, and very rare to variable immunoreactivity for desmin, further supporting the diagnosis of UPS. Fourteen primary tumors were analyzed histologically, and while 13 of these (93%) were UPS, one tumor (7%) was pleomorphic rhabdomyosarcoma (PRMS). Morphologically, the UPS evaluated were very similar to one another in that they demonstrated poorly differentiated cells with marked anaplasia and no line of differentiation by light microscopic appearance. The spectrum of myogenic markers (SMA and desmin) expressed by the UPS is reflected in the pattern of expression in human tumors (i.e. some tumors express one or both of the markers, and some do not). The PRMS arose in an IM-injected animal and was characterized by the presence of myogenin immunoreactivity (Fig 5C). A variable pattern of AKT activation was noted in the tumors analyzed, with phospho-AKT staining diffusely in some tumors, including the PRMS tumor and some of the UPS tumors (Fig 5D, left panel), and more focally in others (Fig 5D, right panel). The sarcoma type and immunohistochemistry profiles for the *Trp53/Pten* knockout tumors are summarized in Table 1.

UPS demonstrated variable immunoreactivity for SMA and desmin, but did not express myogenin. PRMS was defined by immunoreactivity for myogenin. Over 90% of the sarcomas generated by this mouse model were UPS. Additionally, 64% of tumors contained a lymphocytic infiltrate, and 71% of tumors expressed PD-L1. Tum-tumor; Ker-keratin; Des-desmin; Myo-myogenin; Lymph-lymphocytic infiltrate; SQ-subcutaneous; IM-intramuscular; D-diffuse (>66%); V-variable (33–66%); F-focal (5–33%); R-rare (<5%).

### Evidence of immune tolerance in the tumorigenesis of undifferentiated pleomorphic sarcoma

**Fig 6:**
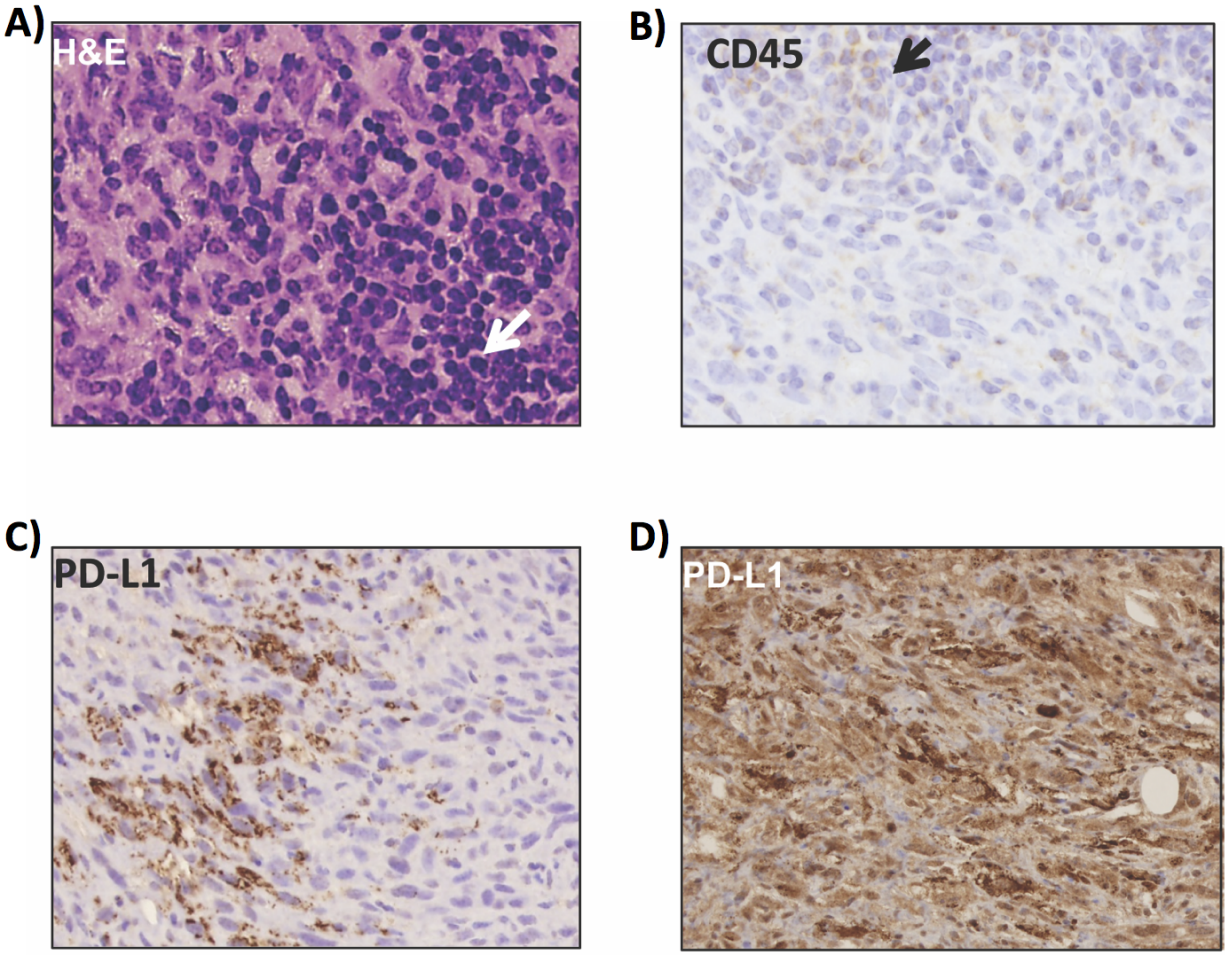
*Trp53/Pten* undifferentiated pleomorphic sarcomas demonstrate immune tolerance. A) Histology of UPS reveals an infiltrate of small mature lymphocytes (arrow), which is further confirmed by immunohistochemistry for CD45 (B, arrow). Immunohistochemistry was performed for PD-L1 (B7-H1), showing expression patterns ranging from focal/variable staining (C), to diffusely and strongly positive (D), suggesting that UPS in the *Trp53/PTEN* model are immunogenic and might evade immunosurveillance by PD-L1 upregulation.

Pathological evaluation revealed an infiltrate of small mature lymphocytes (Fig 6A and Table 1) in 64% of the sarcomas, confirmed by immunoreactivity for CD45 (Fig 6B). The presence of small mature lymphocytes suggested the presence of immune surveillance in the immunocompetent mouse model of UPS. To test the hypothesis that sarcomas in this mouse model demonstrated immune tolerance, we performed immunohistochemistry for PD-L1 (B7-H1) and demonstrated 71% of the tumors to upregulate PD-L1. The pattern of expression ranged from focal to variable (Fig 6C) to diffusely and strongly positive (Fig 6D). No correlation between the presence of a lymphocytic infiltrate and PD-L1 expression was identified. Taken together, the above findings suggest the UPS formed in this mouse model are immunogenic, and that a substantial proportion might evade immunosurveillance by upregulation of PD-L1 and inhibition of T lymphocyte activity [25].

## Discussion

Herein we describe a new mouse model for UPS that incorporates viral Cre-mediated deletion of *Pten/Trp53* and BLI capability. This has the advantage of allowing for the formation of tumors in the extremities where they can be closely monitored. This model is also unique in that Cre recombinase expression and allelic recombination results not only in homozygous loss of *Trp53* and *Pten*, but also in expression of luciferase. Analysis of the TCGA database indicates that combined loss of *PTEN* and *TP53* are more common in human UPS than other soft tissue sarcomas, highlighting the relevance of this model to human disease. BLI allows quantitative measurement of tumor growth earlier, beginning around 1 week post virus injection, than is possible for manual estimation of tumors, which are not palpable until 10–11 weeks. This makes BLI well suited to measure early tumor growth, before it is possible to measure tumor volume via calipers. It is important to note, however, that BLI may not proportionally scale with tumor volume, particularly in larger tumors, due to the presence of non-viable necrotic regions, attenuation of light signal from the interior of the tumor by overlying tissue, and/or variability in intratumoral exposure to luciferin [25–27]. This BLI capability was also instrumental in uncovering inherent differences between SQ and IM injection of Ad-Cre with regards to systemic spread, which likely would not have been realized in the absence of serial BLI. Although tumors arising in SQ and IM locations were histologically and immunohistochemically similar, IM adenoviral injection resulted in greater systemic dissemination and unanticipated infection of Ad-Cre, a phenomenon which may need to be taken into account during experimental design. Abdominal BLI signal was found to be consistent with multifocal liver lesions on *ex vivo* imaging and hepatic extramedullary hematopoiesis on histologic examination.

An additional point to consider with systemic viral dissemination is whether this might be avoided or curtailed with modified viral preparation or use of a different expression promoter. The current study uses the same viral vector (Ad-Cre driven by the CMV promoter) as the Kirsch study, although the method of viral preparation prior to injection differs. In the current study, virus is prepared in phosphate-buffered saline. In the *Trp53/Kras* model, virus is prepared in MEM and CaCl_2_ buffer [18, 28]. This preparation is thought to facilitate the formation of precipitates, which are then taken up into cells via endocytosis [28]. It is possible that viral preparation as a precipitate might decrease absorption into the circulation and increase uptake specifically into the muscle and SQ tissue [28]. The CMV promoter is used to drive Cre recombinase in the present study as well as in the Kirsch study, and although this promoter has the advantages of strong, constitutive expression, it also lacks cell-type specificity. Similar to strategies used to target Cre recombinase expression in other transgenic sarcoma models, it may be possible to engineer Ad expression vectors where Cre is driven under the expression of tissue-specific promoters, therefore targeting allelic recombination to specific cell types. This type of vector engineering, though limited by constraints such as vector size, has the possibility of tailoring this Ad-Cre model for additional applications.

Both the SQ- and IM-injected cohorts developed primary soft tissue sarcomas with 100% incidence and comparable latencies. However, the nature of the tumors on physical exam and the associated animal morbidity and survival were very different between the injection groups. The sarcomas that arose following SQ viral injection were easily identified by physical exam due to their superficial nature and location, which was amenable to palpation. The tumors were rubbery and mobile, and were able to readily grow for long periods of time with limited animal morbidity. The sarcomas that developed following IM injection into the quadriceps were difficult to palpate until they had reached a large size, and accurate caliper measurements were challenging due to the deeply invasive nature of the tumors within the native musculature. The tumors were firm, fixed, and caused significant animal morbidity with decreased ability to bend the hind leg and ambulate, resulting in increased morbidity. Depending on the tumor characteristics desired by the researcher, the injection technique could be tailored for SQ or IM injection to induce either superficial, indolent tumors versus invasive, aggressive tumors, respectively.

Our Trp53^fl/fl^Pten^fl/fl^ model demonstrates that homozygous inactivation of both *Trp53* and *Pten* in either SQ tissue or in skeletal muscle promotes sarcomagenesis. It is unclear whether this occurred because the tumor initiating cell in both anatomic sites is the same, or whether UPS represents a common final pathway for sarcomas with multiple lines of differentiation. One exception in the current study is the development of PRMS in a single intramuscular-injected mouse. Given the small number of mice injected, it would be interesting to expand the power of the study to determine if additional defined sarcoma subtypes might develop in greater frequency if provided the opportunity, such as liposarcomas and rhabdomyosarcomas.

The presence of a lymphocytic infiltrate in the majority of the sarcomas in this immunocompetent mouse model strongly suggests these sarcomas are immunogenic. Expression of PD-L1 in sarcomas generated in this mouse model indicates these tumors grow despite immune surveillance by expression of a check-point protein that inactivates T lymphocytes [29]. These findings suggest that this mouse model can be used as a pre-clinical model to test the efficacy of anti PD-1/PD-L1 therapy (e.g. pembrolizumab) in UPS.

In conclusion, we have developed a novel mouse model of UPS, which is driven by Ad-Cre recombinase mediated inactivation of *Trp53* and *Pten* in either SQ tissue or skeletal muscle. Injection into the extremities offers the ability to closely monitor growth of the tumors. Further facilitating the observation of the tumor is concurrent expression of luciferase following inactivation of *Trp53* and *Pten*, providing the ability to use BLI to track allelic recombination, tumor formation, and tumor growth. This *Trp53/Pten* model has the potential to be further tailored to the researcher’s needs by modifying the viral titer injected, the method of viral preparation, or the anatomic site and timing of injection. The technique of SQ or IM Ad-Cre injection could also be applied to other transgenic/knock-out mice. Additionally, serial *in vivo* BLI of UPS is a useful tool for tracking tumor response to novel therapeutic treatments in pre-clinical models, further providing translational insight into this aggressive neoplasm.

## Supporting information

S1 File Raw BLI and mouse data. (XLSX)

S1 Fig Loss of PTEN expression by UPS tumors. Immunohistochemistry reveals the neoplastic cells to lack expression of PTEN, confirming recombination of both floxed *Pten* alleles. The inset demonstrates the presence of infiltrating inflammatory cells expressing PTEN (internal positive control).
(TIFF)

S2 Fig *Trp53/Pten* mice injected with Ad CMV-eGFP demonstrate no appreciable BLI signal. A) BLI of *Trp53/Pten* mice injected with either IM Ad CMV-eGFP or Ad CMV-Cre were compared over the course of one week. Shown in (B) is quantified whole-body *in vivo* BLI values for mice from each injection group (n = 3). These data demonstrate that while Ad CMV-Cre mice have significant BLI signal, Ad CMV-eGFP mice have no appreciable luciferase expression. Panel (C) shows *ex vivo* BLI of dissected livers from each group, highlighting the source of abdominal BLI in the IM-injected Cre cohort compared to the absence of abdominal liver signal in the control eGFP cohort.
(TIFF)
