# Supplementary figures and images for: A Trp53fl/flPtenfl/fl mouse model of undifferentiated pleomorphic sarcoma mediated by adeno-Cre injection and in vivo bioluminescence imaging
Source: PLoS One. 2017 Aug 25;12(8):e0183469. doi: 10.1371/journal.pone.0183469 (PMC5571905; doi:10.1371/journal.pone.0183469)

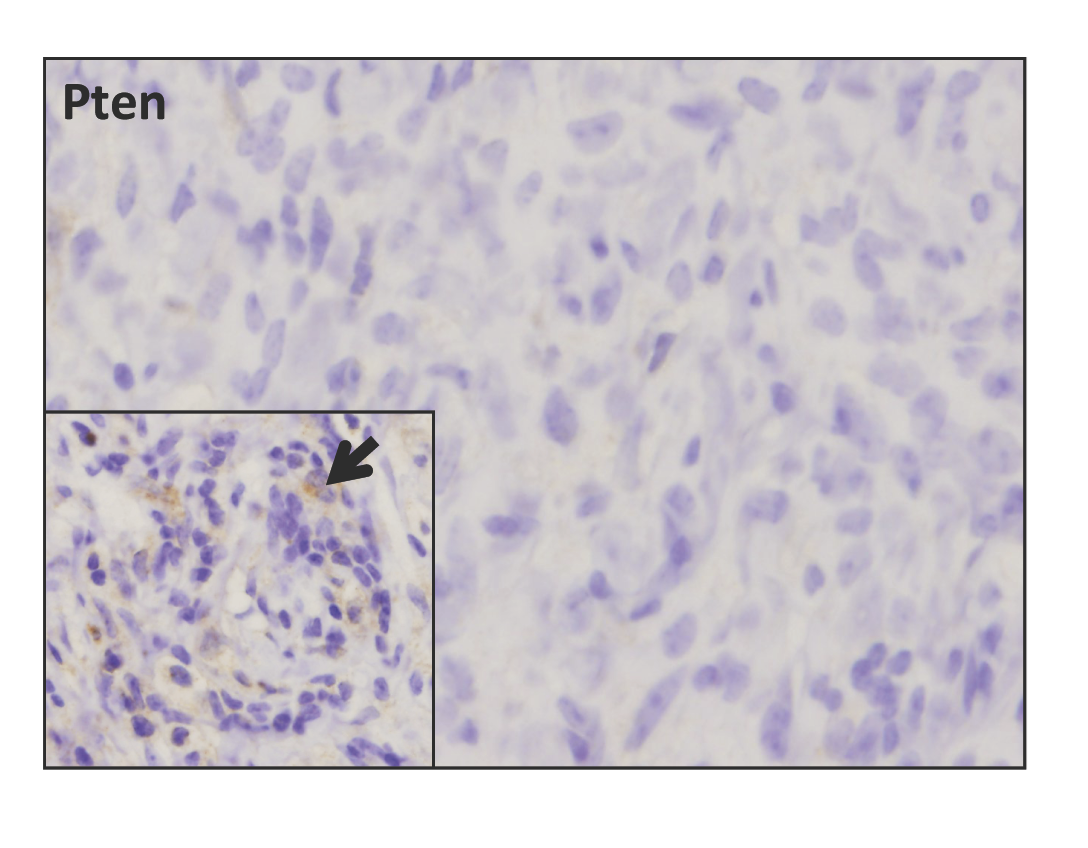

Supplement: S1 Fig — Immunohistochemistry reveals the neoplastic cells to lack expression of PTEN, confirming recombination of both floxed Pten alleles. The inset demonstrates the presence of infiltrating inflammatory cells expressing PTEN (internal positive control). (TIFF) [file pone.0183469.s002.tiff]

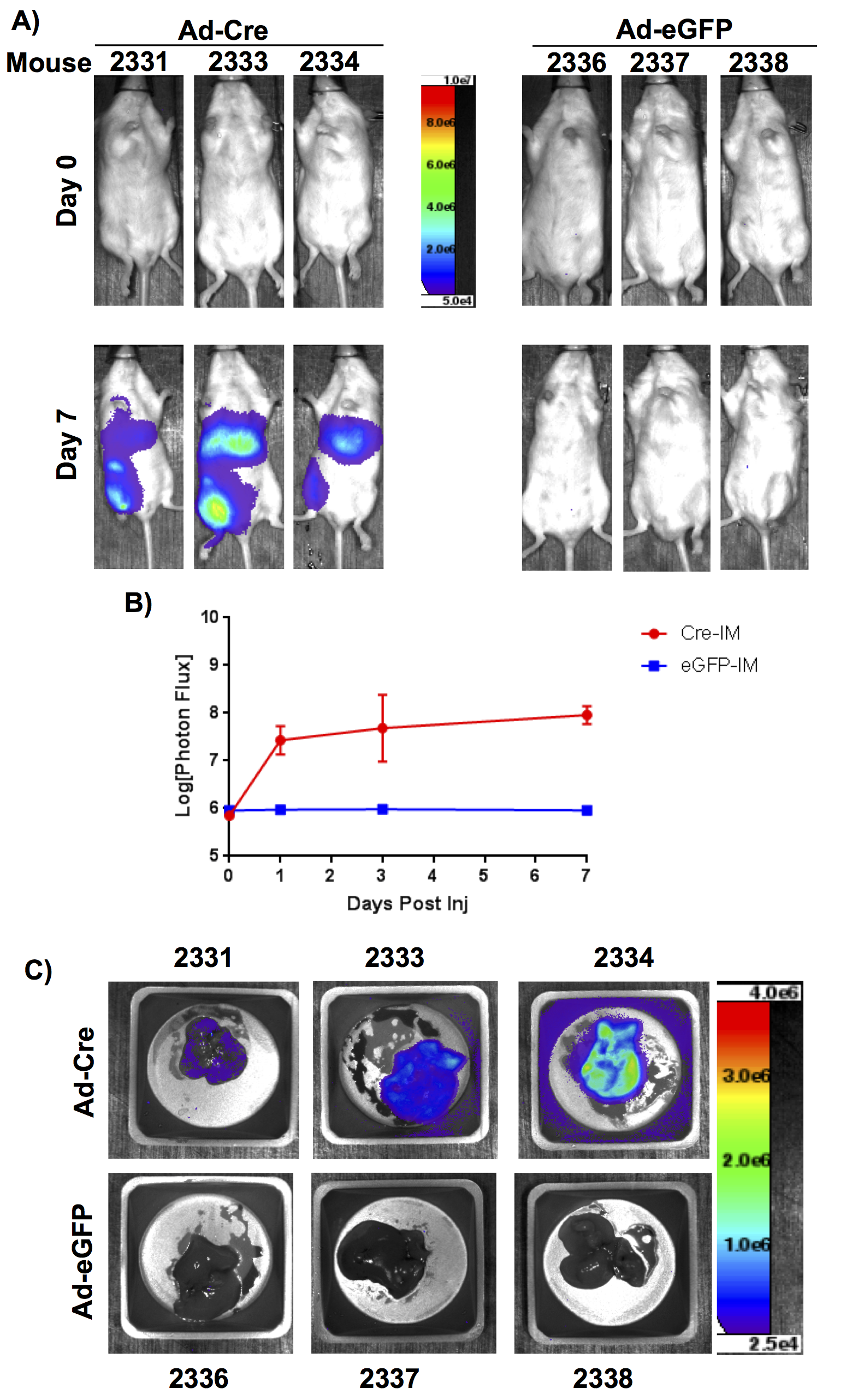

Supplement: S2 Fig — A) BLI of Trp53/Pten mice injected with either IM Ad CMV-eGFP or Ad CMV-Cre were compared over the course of one week. Shown in (B) is quantified whole-body in vivo BLI values for mice from each injection group (n = 3). These data demonstrate that while Ad CMV-Cre mice have significant BLI signal, Ad CMV-eGFP mice have no appreciable luciferase expression. Panel (C) shows ex vivo BLI of dissected livers from each group, highlighting the source of abdominal BLI in the IM-injected Cre cohort compared to the absence of abdominal liver signal in the control eGFP cohort. (TIFF) [file pone.0183469.s003.tiff]
